# STREPTOCOCCUS VIRIDANS NECROTIZING FASCIITIS PRESENTING AS FOURNIER GANGRENE IN A DIABETIC PATIENT

**DOI:** 10.51894/001c.123049

**Published:** 2024-08-30

**Authors:** Aaron Ghandi

**Affiliations:** 1 Internal Medicine Garden City Hospital

## 66

### INTRODUCTION

Necrotizing fasciitis is an infection of the deep soft tissues associated with significant mortality, even when managed aggressively in an Intensive Care Unit. Its incidence ranges from 0.3 to 15 cases per 100,000 individuals. Infection can be monomicrobial or polymicrobial, with polymicrobial infection typically occurring in immunocompromised individuals.

### CASE DESCRIPTION

Our patient had a past medical history of Type 2 diabetes mellitus, hyperlipidemia, and hypertension and presented to the Emergency Department with one week of bowel incontinence which progressed to severe pain associated with perianal erythema, swelling, and warmth. On examination he was found to have a swollen right buttock with erythema and fluctuance extending into the perianal area and right scrotum. Hemoglobin A1C was >14.0%. CT pelvis was originally read as left perianal fistula. High clinical index of suspicion was present for necrotizing fasciitis. Repeat review of imaging completed with Radiology and General Surgery identified subcutaneous gas and stranding consistent with necrotizing fasciitis. Tissue culture revealed heavy growth of viridans streptococcus and moderate growth of streptococcus agalactiae. The patient required intubation, vasopressor support, broad-spectrum antibiotics, aggressive fluid resuscitation, and underwent numerous instances of surgical debridement. Despite aggressive management, clinical status declined, multisystem organ failure was diagnosed, and the patient was compassionate extubated.

### DISCUSSION/CONCLUSIONS

Necrotizing fasciitis of the perineum is known as Fournier gangrene, which typically occurs after disturbance of gastrointestinal mucosa. *Escherichia coli* is the most commonly implicated pathogen. It occurs more commonly in men than women. Factors associated with increased mortality are delay in surgery for more than 24 hours, white blood cell count of over 30,000/microL with band neutrophils greater than 10%, serum creatinine greater than 2.0mg/dL, and age >60. This case illustrates the importance of a high clinical index of suspicion and the benefit of a multidisciplinary approach to care.

